# Transportation noise pollution as a cardiovascular risk factor: from epidemiological evidence to mechanistic insights

**DOI:** 10.17179/excli2025-9050

**Published:** 2025-12-02

**Authors:** Thomas Münzel, Marin Kuntic, Michael Molitor, Mette Sørensen, Andreas Daiber

**Affiliations:** 1 University Medical Center Mainz, Department of Cardiology, Mainz, Germany; 2 Danish Cancer Institute, Danish Cancer Society, Copenhagen, Denmark; 3 Department of Natural Science and Environment, Roskilde University, Roskilde, Denmark

**Keywords:** transportation noise, cardiovascular disease, oxidative stress, endothelial dysfunction, inflammation, environmental risk factors

## Abstract

Transportation noise from road, rail, and aircraft traffic is now recognized as a major cardiovascular risk factor. In Europe, more than 113 million people are chronically exposed to levels above 55 dB(A), resulting in an estimated 1.3 million healthy life-years lost annually from traffic-related noise. Large epidemiological studies consistently demonstrate associations with ischemic heart disease, heart failure, stroke, and type 2 diabetes, with additional links to hypertension, atrial fibrillation, and obesity. Translational and experimental research has clarified the biological plausibility of these findings. The central “noise reaction model” involves activation of the sympathetic nervous system and hypothalamic-pituitary-adrenal axis, with subsequent release of catecholamines and cortisol. These stress responses provoke endothelial dysfunction, vascular inflammation, and oxidative stress, largely through NADPH oxidase 2 activation and nitric oxide synthase uncoupling. At the molecular level, noise alters gene expression networks, disrupts circadian clock regulation, downregulates FOXO3, and induces pro-inflammatory epigenetic modifications. Neuroimaging studies reveal chronic noise activates the amygdala, linking stress perception to vascular inflammation and major adverse cardiovascular events. Adverse effects are most pronounced at night, when noise fragments restorative sleep and amplifies neurohormonal imbalance. Importantly, these pathways overlap with mechanisms of traditional cardiovascular risk factors - diabetes, hypertension, smoking, and hyperlipidemia - suggesting that noise accelerates vascular aging through convergent mechanisms. Combined exposure to noise and air pollution further exerts additive or synergistic effects, underscoring the value of the exposome concept in identifying vulnerable populations. Transportation noise should therefore be considered an established cardiovascular risk factor, requiring equal priority in prevention guidelines and public health policy.

See also the graphical abstract(Fig. 1).

## Introduction

Air pollution is an established risk factor for cardiovascular disease (CVD) (Munzel et al., 2021[81], 2025[83]). Much less attention has been devoted to environmental noise, although both exposures co-exist in urban areas and share similar pathophysiological pathways. Historically regarded as a nuisance or quality-of-life issue, environmental noise is now firmly recognized as a widespread cardiovascular risk factor. The WHO concluded in its 2018 Environmental Noise Guidelines that high-quality evidence links road traffic noise to ischaemic heart disease (WHO, 2018[124]).

**Figure 2:**
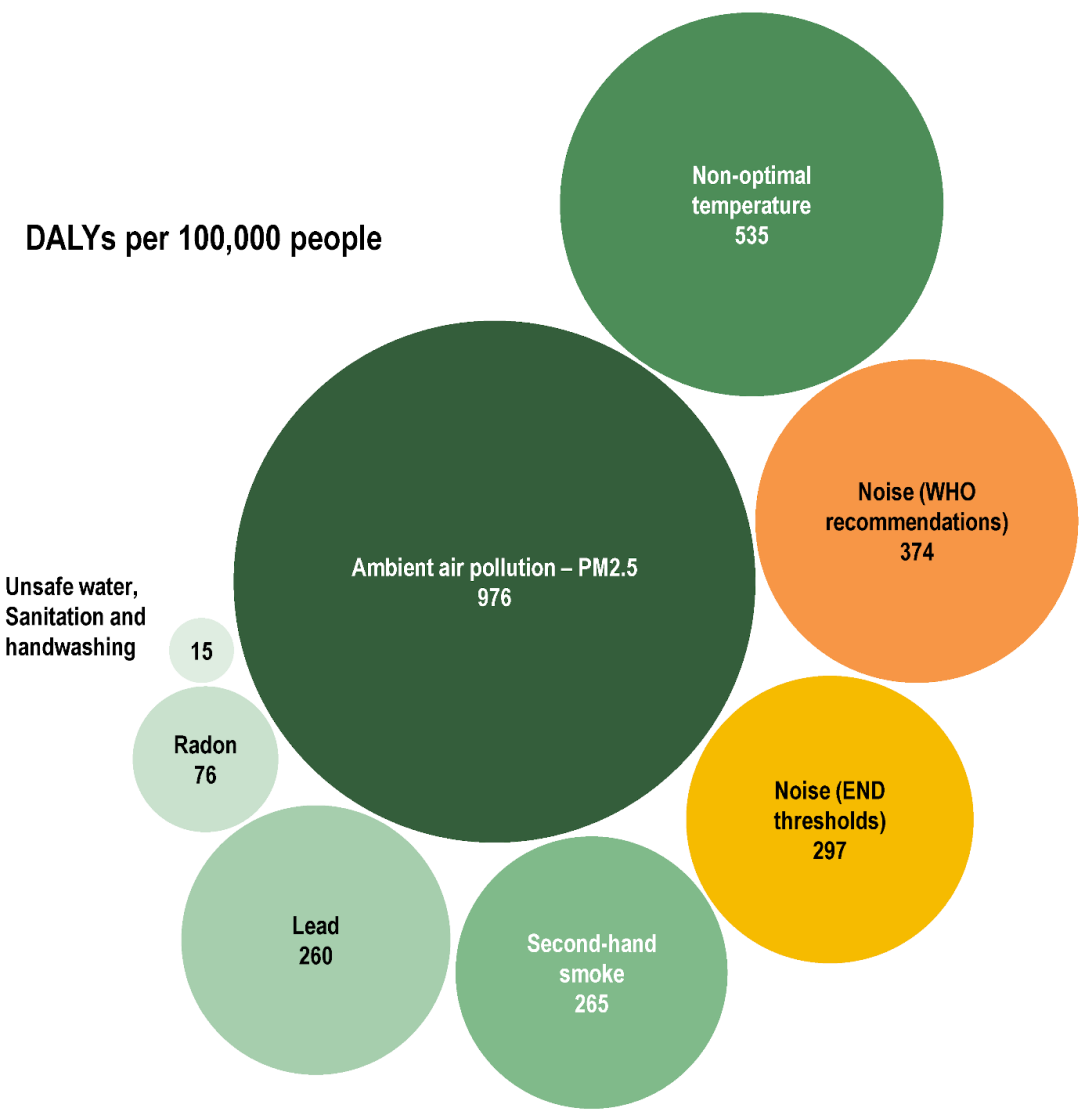
Annual disability-adjusted life years (DALYs) per 100,000 people attributable to selected environmental risk factors in the EEA-32 countries (excluding Türkiye). The bubble plot illustrates the relative burden of different exposures, with bubble size proportional to attributable DALYs. Ambient air pollution (PM2.5) represents the largest contributor (976 DALYs per 100,000), followed by non-optimal temperature (535), environmental noise (374 DALYs at WHO recommendations; 297 DALYs at European Noise Directive (END) thresholds), second-hand smoke (265), lead exposure (260), radon (76), and unsafe water, sanitation and handwashing (15). Colors differentiate environmental and climate risk factors (green/blue tones) from environmental noise (yellow/orange tones). Graph was taken from (EEA, 2025) with permission.

According to the European Environment Agency (EEA, 2025[21]) report, more than 110 million Europeans, over 20 % of the population, are exposed to transportation noise levels exceeding the EU reporting thresholds for health effects, and nearly 150 million exceed the stricter WHO guideline values (EEA, 2025[21]). Chronic exposure to transport noise is estimated to cause at least 66,000 premature deaths, 50,000 new cases of CVD, and 22,000 new cases of type 2 diabetes annually in Europe, resulting in a loss of ~1.3 million disability-adjusted life years (DALYs), placing noise among the top three environmental risk factors contributing to the DALY burden (Figure 2(Fig. 2); Reference in Figure 2: EEA, 2025[21]).

The EEA estimated the economic costs of noise to be at least € 95.6 billion per year, ~0.6 % of the EU's GDP (EEA, 2025[21]). Despite this substantial burden, noise is insufficiently represented in the GBD 2019 Risk Factors Collaborators (2020[32]) and is still absent from major CVD prevention guidelines (Writing Committee Members, 2025[127]). Unlike auditory damage, the cardiovascular effects of noise occur via indirect pathways. The “noise reaction model” describes how the perception of noise particularly during sleep, activates limbic and cortical structures, triggering the hypothalamic-pituitary-adrenal (HPA) axis and sympathetic nervous system (SNS) (Babisch, 2003[1], Munzel et al., 2014[77]). This leads to the release of cortisol, adrenaline, and noradrenaline, which promote endothelial dysfunction, oxidative stress, inflammation, circadian disruption, and vascular remodeling (Munzel et al., 2017[76], 2018[80]). Importantly, these pathophysiological cascades overlap with those triggered by air pollution and other environmental stressors, suggesting additive or synergistic effects (Munzel et al., 2020[78]).

This review integrates epidemiological associations, discusses mainly the mechanistic insights from human and molecular animal studies, and emerging exposome approaches. Drawing on recent umbrella reviews (Blanes et al., 2023[9]) and policy reports (EEA, 2025[21]), we advocate for the recognition of transportation noise as a fully established cardiovascular risk factor and its integration into prevention strategies, urban planning, and clinical practice.

## Historical Milestones in Understanding Noise as a Cardiovascular Risk Factor

Although the detrimental effects of noise were initially recognized in the context of occupational hearing loss, non-auditory health impacts have been documented for more than half a century. In the 1960s, Levi and colleagues demonstrated that industrial noise exposure induced elevations in blood pressure and catecholamine release in workers (Levi, 1967[66]). Shortly thereafter, Kryter proposed that noise should be regarded as a generalized biological stressor capable of provoking systemic physiological changes even at subauditory thresholds (Kryter, 1970[56]).

Animal experiments in the 1970s and 1980s confirmed these concepts: Yamamura and others showed that intermittent and low-frequency noise elicited stronger autonomic and vascular responses than continuous or high-frequency sound (Yamamura et al., 1981[128]). These observations foreshadowed modern insights into noise-induced arousals, sleep fragmentation, and their downstream cardiovascular consequences.

In the late 20th century, Babisch integrated epidemiological and mechanistic evidence into the “noise reaction model,” which describes how cognitive perception of sound activates the HPA axis and SNS, ultimately leading to hypertension, vascular dysfunction, and increased cardiovascular risk (Babisch, 2003[1]).

The past two decades have witnessed a paradigm shift toward recognizing transportation noise as a major environmental determinant of cardiovascular health. Landmark reviews have consolidated epidemiological data, mechanistic human and animal evidence, and translational findings. In particular, the 2014 *European Heart Journal* review provided a comprehensive synthesis of noise-induced vascular damage, oxidative stress, and endothelial dysfunction (Munzel et al., 2014[77]). The 2018 WHO Environmental Noise Guidelines (WHO, 2018[124]) and the 2021 *Nature Reviews Cardiology *article (Munzel et al., 2021[81]) further validated these associations, introducing molecular concepts such as oxidative stress, circadian disruption, and neuroinflammation.

Most recently, the 2025 EEA report quantified the burden at the European level, attributing tens of thousands of premature deaths and new cardiovascular cases annually to traffic noise (EEA, 2025[21]). Together, these milestones have firmly established environmental noise as a cardiovascular risk factor requiring urgent preventive action in clinical and public health practice.

## Epidemiology

The WHO Environmental Noise Guidelines (ENG) identified road traffic noise as a well-established risk factor for ischemic heart disease (IHD) (WHO, 2018[124]). Since then, several large-scale European studies have reinforced these findings (Blanes et al., 2023[9]). A meta-analysis of nine cohorts showed that every 10 dB(A) increase in road traffic noise is associated with a 5 % rise in cardiovascular mortality (Blanes et al., 2023[9]). Acute triggering effects have also been documented: in Switzerland, exposure to aircraft noise within the two hours before death significantly elevated the likelihood of cardiovascular mortality (Saucy et al., 2021[97]).

For IHD specifically, the WHO ENG reported a relative risk (RR) of 1.08 (95 % CI: 1.01-1.15) per 10 dB(A) increase in L_den_ (Kempen et al., 2018[50], WHO, 2018[124]). More recent evidence, including studies on nine pooled Scandinavian cohorts (Pyko et al., 2023[92], Sorensen et al., 2024[106]) and a Danish nationwide study of >2.5 million people (Poulsen et al., 2023[89], Thacher et al., 2022[115]), confirmed the association , with hazard ratios (HRs) for IHD of 1.03-1.05 per 10 dB(A) higher road traffic noise. A meta-analysis that combined the WHO ENG studies with newer cohorts yielded a pooled RR of 1.04 (95 % CI: 1.02-1.06) (Blanes et al., 2023[9]). Results for railway and aircraft noise are less consistent, with pooled estimates close to unity (Blanes et al., 2023[9], Pyko et al., 2023[92], Thacher et al., 2022[115]). Outside Europe, the Nurses' Health Study in the US linked anthropogenic noise exposure to increased coronary heart disease incidence (Roscoe et al., 2023[93]), but methodological differences, such as reliance on total anthropogenic noise at 270 × 270 m resolution instead of source-specific models, limit comparability with European findings (Vienneau et al., 2023[120]).

When the WHO ENG was published, only one prospective cohort study on noise and stroke was published, showing that road traffic noise was associated with a HR of 1.14 (95 % CI: 1.03-1.25) (WHO, 2018[124]). Since then, multiple investigations, including pooled Danish and Swedish cohorts, have confirmed positive associations (Roswall et al., 2021[96], Sorensen et al., 2021[108]).

**Figure 3:**
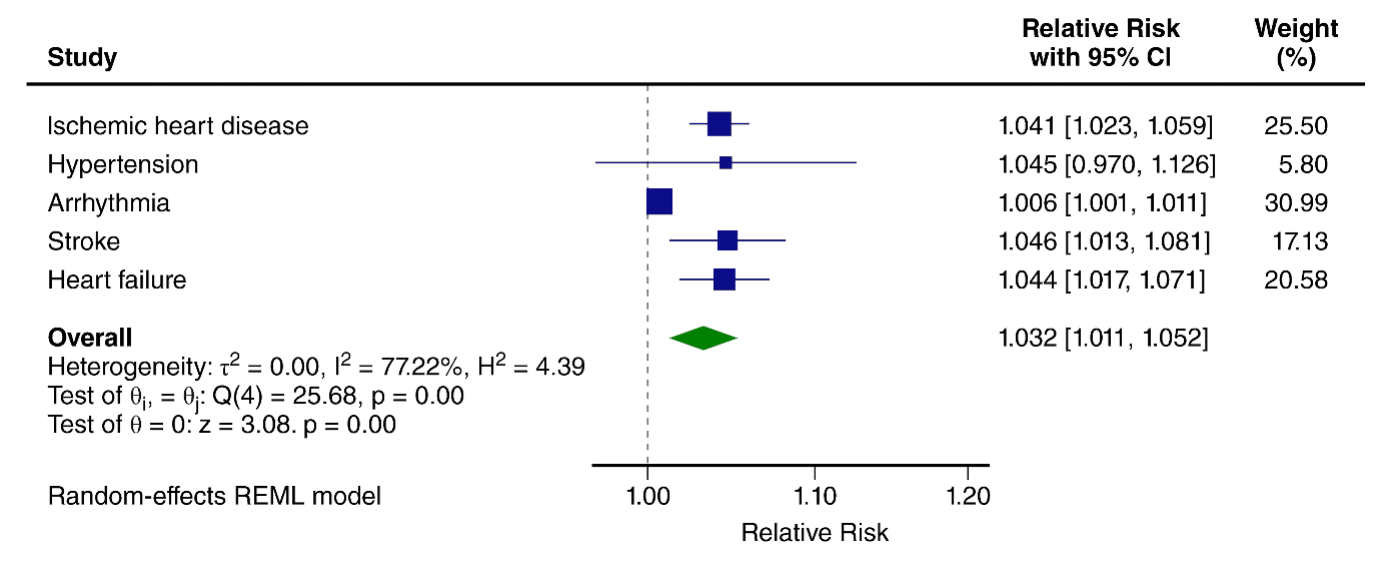
Relative risks obtained in meta-analyses of a Umbrella+ review from 2023 estimating the association between road traffic noise and cardiovascular disease. (Engelmann et al., 2023) In the Umbrella+ review, we conducted a systematic search for the period from 2015 until 2023 and combined with all studies identified until 2015 in a systematic search conducted by a WHO expert group (Engelmann et al., 2023). Relative risks refer to a 10 dB increase in L_den_ (With permission ©European Topic Centre on Human Health and the Environment).

A 2023 umbrella+ meta-analysis, based on six studies, reported a pooled RR of 1.05 (95 % CI: 1.01-1.08) per 10 dB(A) (Figure 3(Fig. 3); Reference in Figure 3: Engelmann et al., 2023[22]) (Blanes et al., 2023[9]). By contrast, associations for railway and aircraft noise remain weak, with pooled RRs close to 1.00 (Blanes et al., 2023[9]).

For heart failure, the WHO ENG did not provide an assessment (WHO, 2018[124]). Since then, at least six longitudinal studies have reported that road traffic noise raises heart failure risk by approximately 4-5 % per 10 dB(A),(Fu et al., 2023[31], Heritier et al., 2017[42], Poulsen et al., 2023[89]) and an Umbrella+ analysis reported a pooled RR of 1.04 (95 % CI: 1.02-1.07) (Blanes et al., 2023[9]). Railway noise was generally not associated with heart failure (Thacher et al., 2022[115]), while aircraft noise results remain inconsistent (Seidler et al., 2016[101], Thacher et al., 2022[115]).

Evidence for arrhythmias, particularly atrial fibrillation, is more limited but points toward weak positive associations. A Danish nationwide cohort study of >3.5 million individuals observed increased risks of atrial fibrillation in relation to road, rail, and aircraft noise (Thacher et al., 2022[114]). The Umbrella+ review estimated that road traffic noise raises arrhythmia risk by ~1 % per 10 dB(A) (RR 1.01, 95 % CI: 1.00-1.01), whereas estimates for aircraft and railway noise remain more uncertain (Blanes et al., 2023[9]).

Beyond incidence, mortality studies highlight the broader burden. Pooled analyses suggest a ~5 % rise in cardiovascular mortality for every 10 dB(A) increase in road traffic noise (Blanes et al., 2023[9]). Furthermore, a Swiss case-crossover study showed that nighttime aircraft noise may trigger fatal cardiovascular events within hours (Saucy et al., 2021[97]).

The lower threshold for harmful noise effects remains uncertain, though it is highly critical input data when conducting health impact assessments. While the EU applies 55 dB(A) L_den_ as the mapping threshold, the WHO recommends 53 dB(A) for road, 54 dB for rail, and 45 dB for air traffic noise (WHO, 2018[124]). Large-scale cohort studies suggest that adverse effects may occur at levels as low as 35-40 dB(A) for stroke (Roswall et al., 2021[96], Sorensen et al., 2021[108]), diabetes (Thacher et al., 2021[113]), and cardiovascular mortality (Sorensen et al., 2024[106], Vienneau et al., 2022[119]), with IHD thresholds estimated between 50 and 55 dB(A) (Pyko et al., 2023[92], Thacher et al., 2022[115]). Health impact modelling in Denmark and Switzerland showed that adopting a lower threshold (45 dB(A) instead of 55 dB(A)) led to three- to four-fold higher estimates of noise-attributable IHD and CVD deaths (FOEN, 2023[26], Thacher et al., 2020[112]).

In summary, the evidence now firmly supports road traffic noise as an environmental risk factor for IHD, stroke, heart failure, and cardiovascular mortality (Blanes et al., 2023[9]). Associations are most consistent for road traffic, while railway and aircraft noise effects are less robust, likely reflecting exposure misclassification and dominance of road noise in urban settings. Despite strong epidemiological support, there remains a lack of high-quality intervention studies showing reduced cardiovascular risk after noise mitigation. Expanding evidence beyond Europe and testing policy-relevant noise reduction strategies remain essential priorities for future research.

## Subclinical Cardiac Remodeling: Insights from CMR

**Figure 4:**
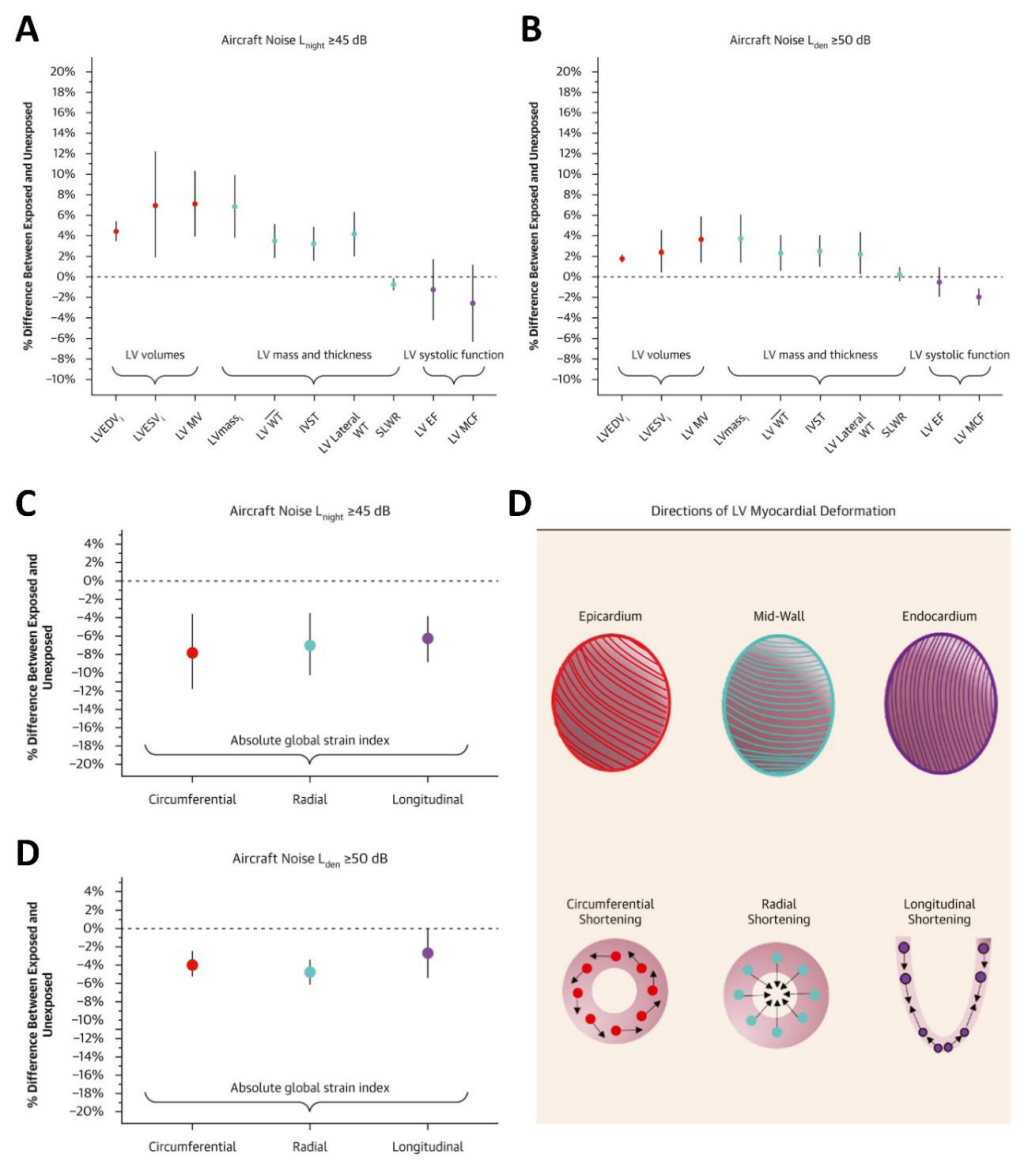
Structural and functional changes of the left ventricle associated with aircraft noise exposure. (A) Individuals exposed to nighttime aircraft noise ≥45 dB (L_night_) showed significant increases in left ventricular (LV) volumes, LV mass and wall thickness, whereas LV systolic function parameters (LV ejection fraction, LV myocardial contraction fraction) were reduced compared with unexposed participants. (B) Similar structural changes were observed for daytime-evening-night noise exposure ≥50 dB (L_den_), with consistent increases in LV volumes and mass and reductions in systolic function. (C, D) Functional myocardial impairment was evidenced by significantly lower global strain indices across circumferential, radial, and longitudinal deformation in participants exposed to ≥45 dB L_night_ (C) and ≥50 dB L_den_ (D). (E) Schematic illustration of left ventricular myocardial deformation: the epicardium, mid-wall, and endocardium contribute to coordinated circumferential, radial, and longitudinal shortening. Noise-exposed participants demonstrated reduced strain across all deformation directions, indicating subclinical myocardial dysfunction. Reproduced from (Topriceanu et al., 2025) with permission.

Beyond clinical endpoints, recent imaging studies provide evidence for subclinical cardiovascular effects of noise (Figure 4(Fig. 4); Reference in Figure 4: Topriceanu et al., 2025[117]). In a landmark JACC publication, Topriceanu et al. linked higher aircraft noise exposure to adverse structural and functional cardiac remodelling assessed by cardiovascular magnetic resonance (CMR) in >3,600 UK Biobank participants. Nighttime aircraft noise (L_night_ ≥45 dB) was associated with ~7 % higher LV mass, ~4 % greater wall thickness, and ~8 % lower global circumferential strain, phenotypes known to predict incident heart failure and major adverse cardiovascular events (Topriceanu et al., 2025[117]). Mediation analyses suggested partial contributions from BMI and hypertension, while non-mover cohorts exhibited more pronounced effects, highlighting cumulative exposure.

In an accompanying editorial, Münzel et al. emphasized that these imaging findings provide crucial mechanistic evidence bridging epidemiology and pathophysiology, underscoring the particular harm of nighttime aircraft noise and calling for urgent policy action (Munzel et al., 2024[79]).

## Beyond Hearing: How Noise Drives Hypertension, Metabolic Dysfunction, and Mental Illness

The idea that noise exerts health effects beyond hearing loss dates back to Kryter's 1970 monograph (Kryter, 1970[57]), which cited early studies showing vasoconstriction and altered hemodynamics during acute noise exposure (Jansen, 1964[46]). By the late 1960s, German industrial workers exposed to loud environments displayed higher rates of circulatory and heart problems (Jansen, 1968[47]). The Speedwell study in 1993 extended this concept to road traffic noise, reporting associations with triglycerides, glucose, blood viscosity, and blood pressure (Babisch et al., 1993[2]), findings later confirmed in experimental and epidemiological studies (Dratva et al., 2011[19], Haralabidis et al., 2008[38]). Occupational studies further showed that high noise (>80 dB[A]) elevates blood pressure, oxidative stress markers, and DNA damage (Bagheri Hosseinabadi et al., 2019[3]).

Hypertension has been most intensively studied. A 2021 review found null results in longitudinal analyses but a 9 % increase per 10 dB(A) in cross-sectional studies (Sivakumaran et al., 2022[105]). Since then, evidence on aircraft noise has been mixed, with US studies showing no association (Kim et al., 2022[53], Nguyen et al., 2023[84]), whereas a French cohort reported increased risk (Kourieh et al., 2022[54]). A 2023 Umbrella+ review found pooled RRs of 1.04 for road and 1.03 for aircraft noise (Blanes et al., 2023[9]), suggesting modest risk increases but underscoring the need for more prospective data.

Noise has also been associated with higher risk of diabetes and obesity, which are key cardiometabolic comorbidities (Buddeke et al., 2019[11], Powell-Wiley et al., 2021[90]). Cohort studies consistently linked road traffic noise to type 2 diabetes (Clark et al., 2017[16], Dimakopoulou et al., 2017[18], Eze et al., 2017[23], Jorgensen et al., 2019[48], Ohlwein et al., 2019[85], Roswall et al., 2018[95], Shin et al., 2020[104], Thacher et al., 2021[113]), with a pooled RR of 1.06 per 10 dB(A) (Liu et al., 2023[67]). Effects are stronger at silent façades (considered a proxy for bedroom exposure) (Sørensen et al., 2023[109] , Thacher et al., 2021[113] ), implicating sleep disturbance as a mediator. Studies also suggest associations with adiposity across the life course, from children to older adults (Christensen et al., 2015[15], Foraster et al., 2018[29], Pyko et al., 2017[91], Sørensen et al., 2022[107], Wallas et al., 2019[122]).

Mental health may also mediate cardiovascular effects. A 2020 meta-analysis reported that road and aircraft noise increase depression risk (Hegewald et al., 2020[39]). Subsequent longitudinal studies linked road traffic noise to depression, reduced well-being, and suicide (Eze et al., 2020[24], Shi et al., 2023[103], Wicki et al., 2023[125]). A landmark BMJ study by Cantuaria et al. showed that long-term exposure to road traffic noise is associated with an elevated risk of dementia, particularly Alzheimer's disease (Cantuaria et al., 2021[13]). Noise annoyance itself predicts later depression and anxiety, highlighting that subjective responses can be early indicators of chronic mental health outcomes (Beutel et al., 2016[8]). Lifestyle changes induced by stress and poor sleep may contribute to poor mental health. Some studies suggest that road traffic noise reduces physical activity (Foraster et al., 2016[28], Roswall et al., 2017[94]) or relates to smoking and alcohol intake (Roswall et al., 2018[95]), though prospective evidence is weak.

Sleep disturbance is among the most consistent pathways. Nighttime noise fragments deep and REM sleep (Basner et al., 2011[5], 2014[4]), prevents blood pressure dipping (Haralabidis et al., 2008[38]), and impairs endothelial function after only one night of exposure (Munzel et al., 2020[78], Schmidt et al., 2015[98]). Intermittent nocturnal noise appears especially harmful (Kroller-Schon et al., 2018[55]). A Zurich case-crossover study confirmed that aircraft noise two hours before death increases CVD mortality risk (Saucy et al., 2021[97]).

Finally, noise annoyance, shaped by exposure intensity, context, and individual sensitivity, remains a sensitive early marker of adverse health effects (Brink et al., 2019[10], Guski, 1999[35], Guski et al., 2017[36]). Annoyance has historically guided policy, and epidemiological evidence increasingly links it to cardiometabolic outcomes (Peeters and Nusselder, 2019[88]).

## Echoes in the Endothelium: Human Mechanistic Studies of Transportation Noise

A series of translational field studies demonstrated that nighttime aircraft and railway noise impairs vascular function, sleep quality, and stress regulation in both healthy volunteers and CVD patients (Schmidt et al., 2013[99], 2015[98]). In these experiments, noise recorded near Düsseldorf airport was replayed in participants' bedrooms (30 or 60 take-off or landing events/night). Even a single night of aircraft noise (L_eq_ 46 dB(A), peak 60 dB(A)) reduced sleep quality, elevated adrenaline, impaired flow-mediated dilation (FMD), and shortened pulse transit time, reflecting sympathetic activation. Notably, acute vitamin C (2 g) restored FMD, implicating oxidative stress. Prior exposure amplified these effects, indicating priming rather than habituation. In line with this, oxidative stress markers such as 3-nitrotyrosine and 8-isoprostane increased significantly (Kroller-Schon et al., 2018[55]).

Effects were stronger in patients with coronary artery disease, suggesting greater vulnerability of already compromised endothelium (Schmidt et al., 2015[98]). Similar findings were reported with nocturnal train noise (30 or 60 train passing events/night, L_Aeq_ 33-54 dB(A)), which also impaired FMD and reduced sleep quality. Vitamin C again mitigated endothelial dysfunction, and proteomics revealed activation of redox, inflammatory, pro-thrombotic, and fibrotic pathways (Herzog et al., 2019[43]).

We further tested whether noise event intensity or frequency differentially affect vascular health. CAD patients exposed to two nighttime aircraft noise scenarios-with equal mean L_eq_ (~45 dB(A)) but differing in event loudness and frequency-showed similar impairments in FMD (Schmidt et al., 2021[100]). Echocardiography revealed diastolic dysfunction (increased E/E′ ratio), and protein assays indicated downregulation of follistatin, glyoxalase I, and ACE-2, linking noise to oxidative stress, inflammation, fibrosis, and cardiac dysfunction.

Human studies indicate that transportation noise affects immune regulation and epigenetic pathways. Cross-sectional cohorts reported elevated IL-12 and high-sensitivity C-reactive protein levels, alongside reduced natural killer cell activity in individuals exposed to noise (Cai et al., 2017[12], Kim et al., 2017[52]), though findings are not fully consistent (Thiesse et al., 2020[116]). These immune alterations have been linked to increased cortisol and greater noise sensitivity, likely reflecting stress-induced HPA axis activation and circadian disruption (Ising and Ising, 2002[45], Selander et al., 2009[102]).

Epigenetic changes provide further support. In the Swiss SAPALDIA cohort, chronic exposure to transportation noise and air pollution produced distinct and overlapping DNA methylation patterns enriched in inflammation, immune response, and cellular development (Eze et al., 2020[24]) pathways. Vascular consequences were also documented: long-term exposure to nocturnal train and road noise increased arterial stiffness, measured by pulse wave velocity (Foraster et al., 2017[27]), while a German study showed that nighttime road traffic noise promoted subclinical atherosclerosis, particularly in individuals with early arterial calcification (Hennig et al., 2020[41], Kalsch et al., 2014[49]).

Together, these findings highlight noise-induced stress signaling, inflammation, oxidative stress, and vascular dysfunction in humans, consistent with mechanistic data from animal studies.

## From Noise to ROS: Animal Models Reveal the Molecular Cascade

**Figure 5:**
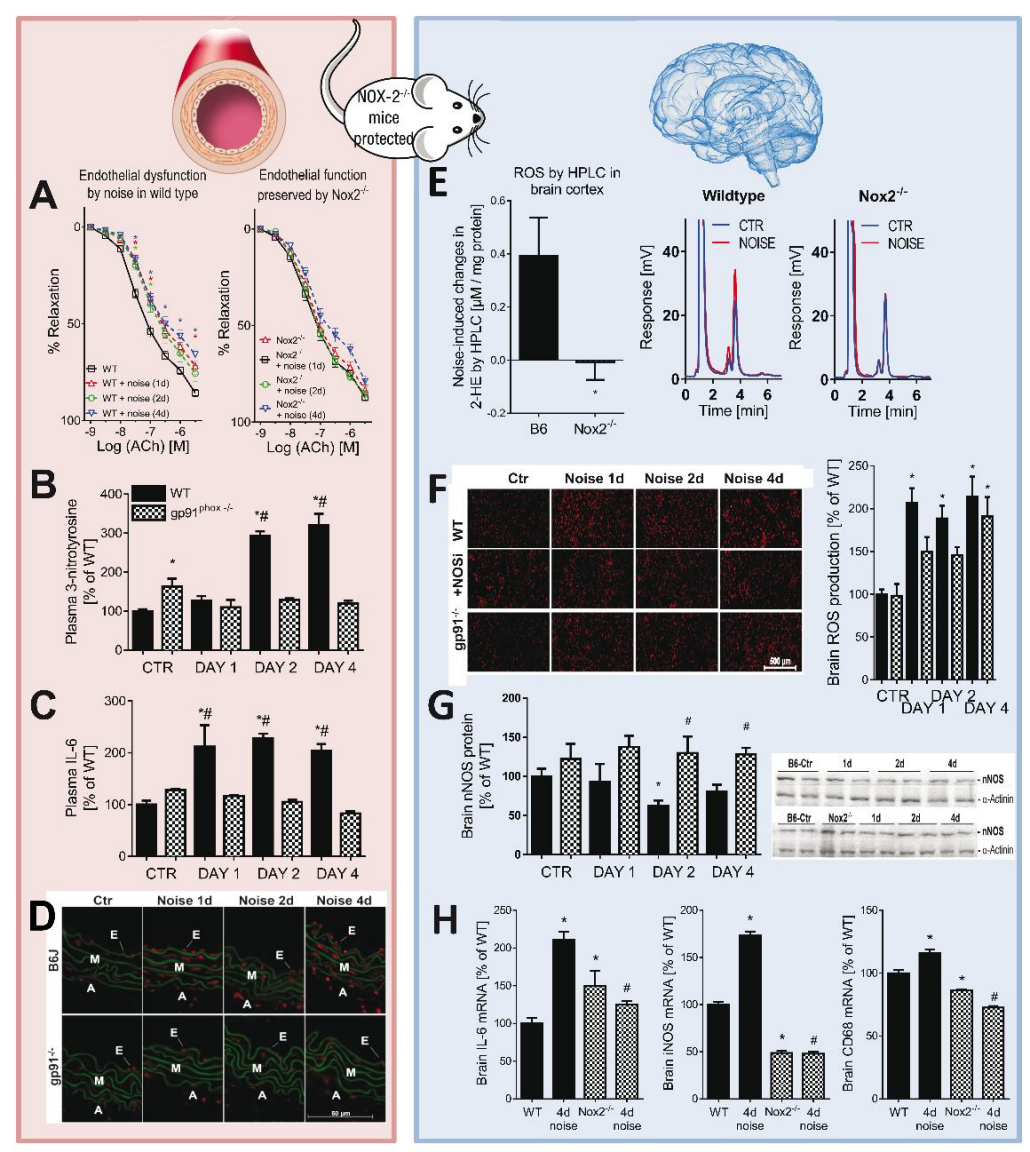
At the vascular level, Nox2-deficient mice were protected against endothelial dysfunction (A), systemic oxidative stress (B), inflammation (C), and aortic ROS generation (D) following aircraft noise exposure (mean sound pressure level 72 dB(A), 24 h/day for 1, 2, or 4 days). At the cerebral level, Nox2 deficiency conferred protection from noise-induced cerebral superoxide and ROS formation, partly arising from uncoupled neuronal nitric-oxide synthase (nNOS) (E,F), as well as from nNOS down-regulation (G) and neuroinflammation (H). Adapted from data in (Kroller-Schon et al., 2018) and reproduced from (Munzel et al., 2020) with permission.

Using a validated murine aircraft-noise paradigm (L_eq_ 72 dB(A), peaks 85 dB(A), up to 24 h/day), we consistently observed rapid neurohumoral activation with rises in stress hormones and blood pressure, coupled to endothelial dysfunction and pronounced oxidative stress in vascular and cerebral tissues (Munzel et al., 2017[76]). Mechanistically, phagocytic NADPH oxidase (NOX-2)-driven reactive oxygen species (ROS) formation emerged as the central hub, reducing nitric-oxide (^•^NO) bioavailability and augmenting superoxide and endothelin-1 expression, and vasoconstrictor sensitivity (Figure 5(Fig. 5); References in Figure 5: Kroller-Schon et al., 2018[55]; Munzel et al., 2020[78]); strikingly, endothelial ^•^NO synthase (eNOS) expression was upregulated yet uncoupled via S-glutathionylation, a known route to impaired ^•^NO signaling (Chen et al., 2010[14], Kroller-Schon et al., 2018[55], Münzel, 2017[75]).

White noise of similar average intensity failed to reproduce these effects, underscoring that spectral content and event pattern, not mean level alone, dictate pathological effects (Munzel et al., 2017[76]). Complementary evidence included oxidative DNA damage and increased NOX-2 expression after 4 days of exposure, with Ogg1-/- mice displaying amplified leukocyte oxidative bursts and inflammatory signatures (Kvandova et al., 2020[61]).

Microvascular readouts linked this inflammatory oxidative milieu to functional constriction: aircraft noise increased leukocyte adhesion and reduced microvascular diameter, red blood cell velocity, and segmental flow changes prevented by genetic NOX-2 deletion (gp91phox) (Eckrich et al., 2021[20]). These pathways mirror those of diabetes, hypertension, and smoking (Heitzer et al., 2000[40], Hink et al., 2001[44], Mollnau et al., 2002[74]) and are exacerbated in angiotensin-II-hypertensive mice, which show heightened vascular inflammation, neuroinflammation, and cerebral ROS under noise (Steven et al., 2020[111]).

Complementing the functional vascular data, next-generation sequencing (NGS) of aortic tissue revealed a broad transcriptional reprogramming: 224 genes were differentially expressed (majority down-regulated) with temporal fluctuations across days 1-4 (Munzel et al., 2017[76]). The strongest up-regulated transcripts included Zbtb44, Setad4, Ypel2 and Ihh, whereas Sacs, Nbeal1, PTPN4 and NR4A3 were among the most strongly reduced. Gene-ontology and pathway analyses pointed to perturbations in VSMC contraction, TGF-β/Smad and NF-κB signalling, adrenergic transduction, focal adhesion, cell-cycle control and apoptosis, and, centrally, FOXO-centred stress-adaptation pathways. These NGS signatures mechanistically link the observed NOX-2 upregulation, eNOS S-glutathionylation/eNOS uncoupling, increased endothelin-1 and vascular immune cell infiltration to impaired oxidative-stress resilience, reduced DNA-repair/FOXO activity and enhanced pro-apoptotic programming, and they nominate FOXO, TGF-β/Smad and several novel 'on/off' stress-response genes as candidate targets for validation and intervention (Munzel et al., 2017[76]). A more recent study revealed that short-term aircraft noise stress induces a fundamental metabolic shift in heart proteome and metabolome that bears the hallmarks of cardiovascular disease (Marques et al., 2025[68]). Within 4 days of noise exposure, the heart proteome and metabolome bear the hallmarks of reduced potential for generating ATP from fatty-acid beta-oxidation, the tricarboxylic acid cycle, and the electron transport chain. Overall, a compensatory shift of energy production towards anaerobic glycolysis under noise exposure was observed similar to the metabolic phenotype found in failing and ischaemic hearts. The adverse metabolic rewiring is likely driven by reactive oxygen species (ROS) since the genetic knockout of NOX-2 (gp91phox), a potent source of ROS in phagocytes, was highly protective.

**Figure 6:**
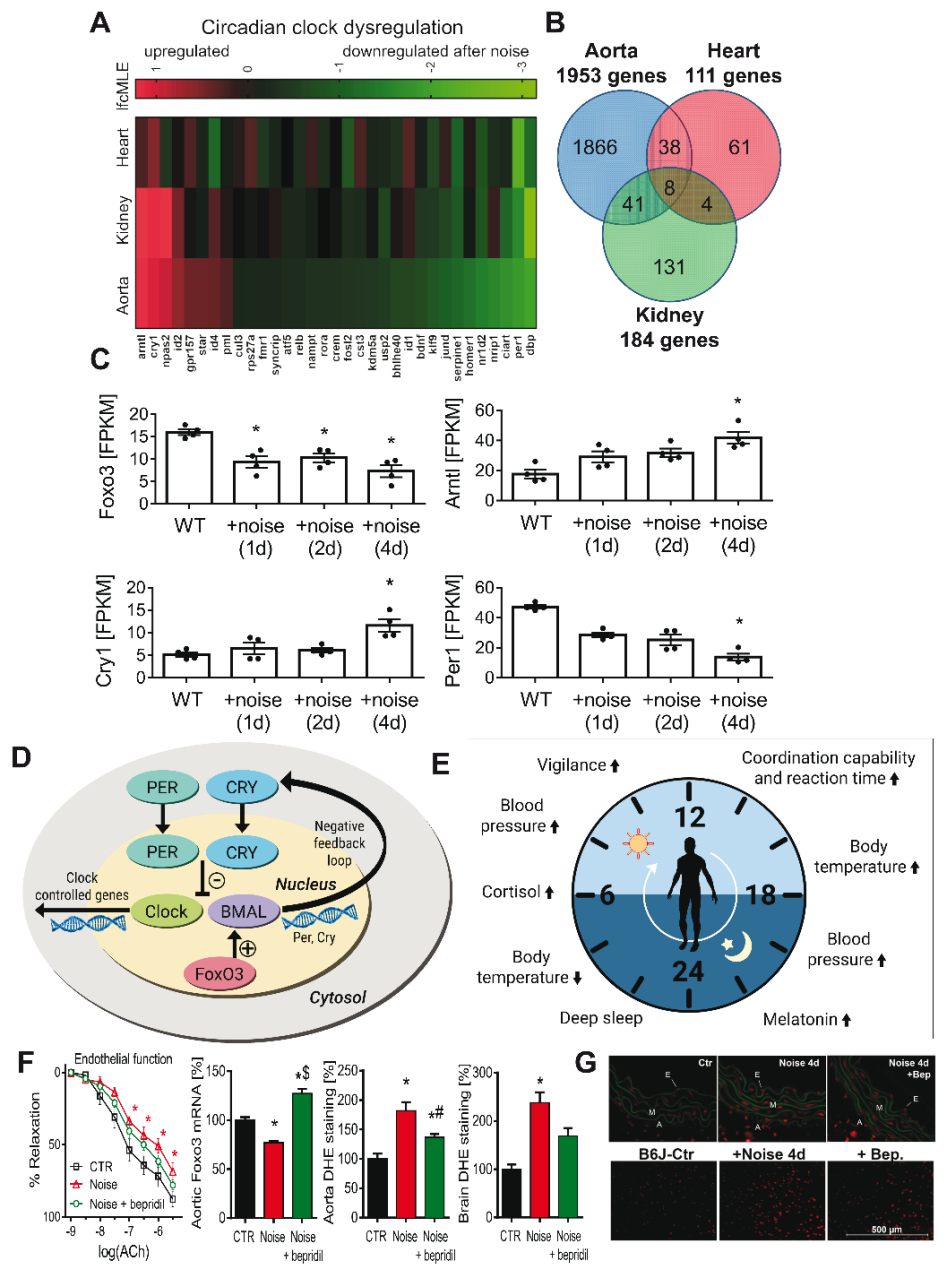
Adverse effects of aircraft noise on circadian regulation. Exposure to aircraft noise (mean sound pressure level 72 dB(A), 12 h/day for 1, 2, or 4 days) induced widespread dysregulation of gene expression in the aorta, heart, and kidney, including circadian clock genes in the aorta and kidney, as revealed by Illumina RNA sequencing (A,B). Specifically, noise down-regulated aortic expression of the transcription factor FOXO3 and period-1 (Per1), while up-regulating brain and muscle aryl hydrocarbon receptor nuclear translocator-like 1 (Bmal1) and cryptochrome-1 (Cry1) (C). FOXO3 interacts with BMAL1 and thereby contributes to circadian rhythm regulation. The circadian clock core consists of the positive regulators CLOCK and BMAL that activate circadian gene expression, and the negative regulators PER and CRY that repress it (D). This molecular clock orchestrates key biological functions, including sleep, body temperature, appetite, and cognition, via time-dependent hormone release such as cortisol and melatonin (E). Pharmacological activation of FOXO3 by the calcium antagonist and antianginal agent bepridil improved endothelial function, normalized FOXO3 mRNA expression, and markedly reduced vascular and cerebral oxidative stress in noise-exposed mice (F). Cytosolic ROS in cryosections of the aorta and frontal cortex was detected by dihydroethidium staining (1 µM) (G). Parts A-C and F-G adapted from (Kroller-Schon et al., 2018) with permission. Part D created de novo and modified from (Kroller-Schon et al., 2018). Part E created de novo and modified from (Van Laake et al., 2018), original source: www.nobelprize.org. Graph was reproduced from (Munzel et al., 2020) with permission.

When exposure coincided with the sleep phase, injury intensified: endothelial dysfunction, stronger pressor responses, neurohormonal surges, endothelin-1 induction, and widespread oxidative stress were accompanied by dysregulated FOXO3/circadian programs (Figure 6(Fig. 6); References in Figure 6: Kroller-Schon et al., 2018[55]; Munzel et al., 2020[78]; Van Laake et al., 2018[118]); NOX-2 deletion mitigated these effects, while neuronal ^•^NO synthase (nNOS) became downregulated/uncoupled, fostering a neuroinflammatory phenotype that aligns with adverse cognitive impacts seen in aircraft-noise-exposed children (Kroller-Schon et al., 2018[55], Stansfeld et al., 2005[110]).

Chronic experiments dispelled the notion of habituation: up to 28 days of exposure produced sustained endothelial dysfunction, hypertension, and inflammation with progressive vascular/cerebral ROS; whole-blood oxidative bursts peaked at days 4-7, Nos3 and FOXO3 declined in brain, and Vcam1 rose across groups (Frenis et al., 2021[30]). Moreover, short-term noise preconditioned the heart to ischemic injury: before left anterior descending artery (LAD) ligation, mice developed a myeloid pro-inflammatory program (adhesion/diapedesis, ↑Nox2, ↑NF-κB phosphorylation), and after MI they exhibited worse LV function, higher mitochondrial O_2_^• -^, and stronger cytokine responses (IL-6, IL-1β, CCL-2), together with aggravated endothelial dysfunction/vascular ROS, effects abrogated by LysM+ myelomonocytic cell depletion (Frenis et al., 2021[30], Molitor et al., 2023[73]).

Translationally, Gutenberg Health Study data linked higher noise/annoyance to elevated baseline C-reactive pro and larger post-MI LVEF drops, and aircraft noise predicted recurrent events and all-cause mortality in acute coronary syndrome (ACS) patients (HR 1.24 per 10 dB L_den_; combined endpoint HR 1.31) (Molitor et al., 2023[73], Olbrich et al., 2023[86]). Recovery kinetics were compartment-specific: after 4 days of noise, 4 days of cessation normalized aortic endothelium-dependent relaxation and inflammatory markers, whereas cerebral microvascular dysfunction and neuroinflammation persisted, indicating slower restitution of resistance-vessel beds (Bayo Jimenez et al., 2023[7]).

Against this mechanistic backdrop, targeting adrenergic signaling was tested as a focused countermeasure. In C57BL/6 mice exposed to aircraft noise (mean 72 dB(A), peaks 85 dB(A), 4 days), propranolol (15 mg/kg/day s.c.) or phenoxybenzamine (1.5 mg/kg/day s.c.), started one day prior, restored endothelial function in the aorta (normalized ACh responses; unchanged NTG curves) and in pressurized cerebral arterioles while suppressing ROS in both beds (dihydroethidium staining) and reducing cardiac/brain markers of oxidative stress and inflammation (NOX2/NOX1, phospho-p47phox, phospho-MARCKS, IL-6, VCAM-1, CD68; circulating/tissue MCP-1); nNOS downregulation persisted (Kuntic et al., 2025[59]). Systemically, noise increased systolic (and by trend diastolic) BP and significantly raised pulse pressure; α/β-blockade did not normalize SBP/DBP but tended to lower pulse pressure, suggesting improved arterial properties without full hemodynamic reversal.

**Figure 7:**
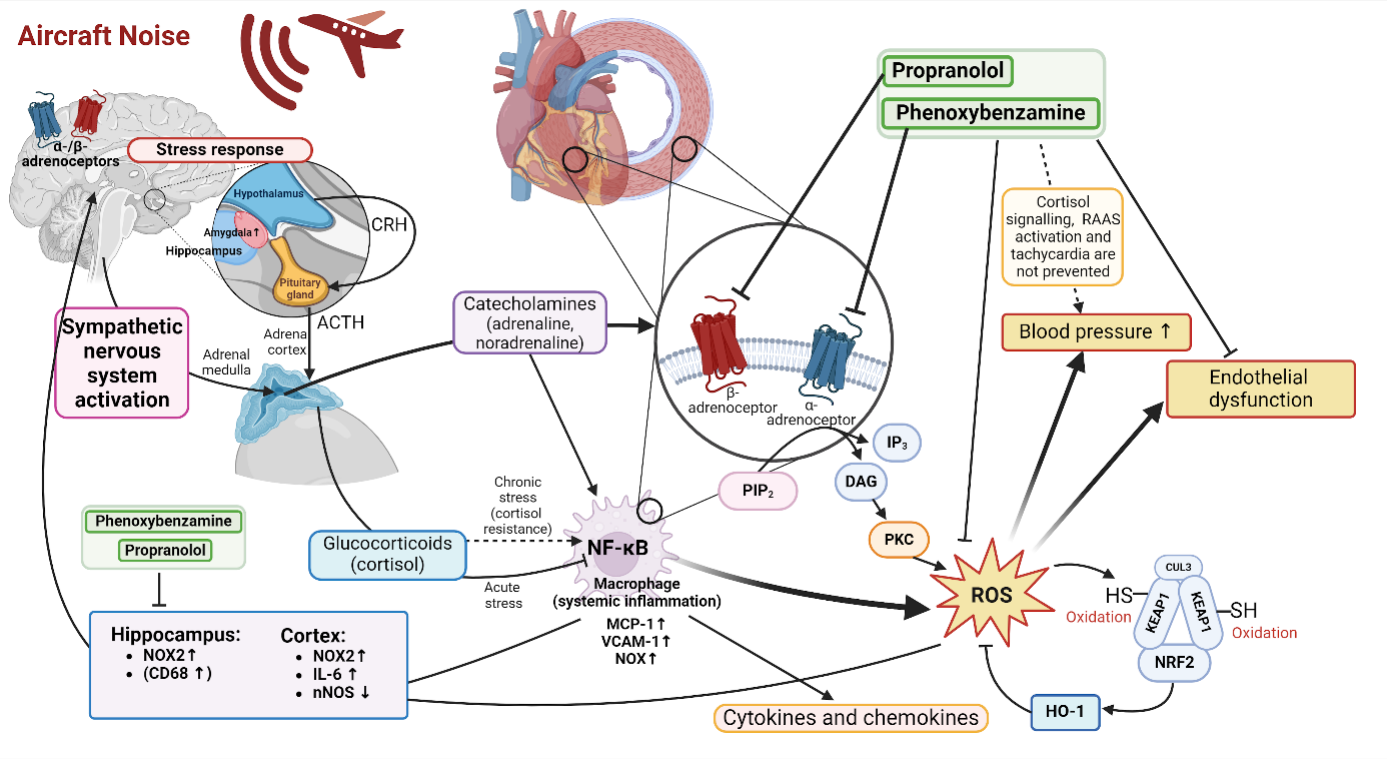
Proposed mechanisms linking aircraft noise exposure to cardiovascular dysfunction. Aircraft noise activates central stress pathways via the amygdala, hippocampus, and hypothalamic-pituitary-adrenal (HPA) axis, leading to sympathetic nervous system activation and release of catecholamines (adrenaline, noradrenaline) and glucocorticoids (cortisol). These stress mediators act on α- and β-adrenergic receptors, stimulating downstream signaling (PIP2, IP3, DAG, PKC) that promotes systemic inflammation (NF-κB activation, macrophage recruitment, cytokine release) and oxidative stress (ROS generation). Elevated ROS induces endothelial dysfunction, vascular inflammation, and blood pressure increases. The pathway also involves chronic cortisol resistance and RAAS activation. Pharmacological blockade with propranolol (β-blocker) and phenoxybenzamine (α-blocker) attenuates some but not all responses, as cortisol signaling, RAAS activation, and tachycardia remain unaffected. Reused from (Kuntic et al., 2025) with permission.

Overall, interrupting SNS outflow at α/β-receptors blunted NOX-2 centric oxidative/inflammatory cascades and rescued endothelial function in conduit and cerebral resistance vessels (Figure 7(Fig. 7); Reference in Figure 7: Kuntic et al., 2025[59]), aligning with prior evidence that catecholamine signaling is a key upstream driver of noise toxicity (Daiber et al., 2020[17], Kroller-Schon et al., 2018[55], Munzel et al., 2017[76]). In parallel, α1AMPK activation via exercise, intermittent fasting, or AICAR robustly prevented endothelial dysfunction and oxidative stress across multiple beds; endothelium-specific α1AMPK loss aggravated injury and abrogated protection, positioning this pathway as a promising lifestyle/pharmacologic lever alongside adrenergic blockade (Kvandova et al., 2023[62], Munzel et al., 2021[81], Visseren et al., 2021[121]). Likewise, pharmacological activation of the Nrf2-heme oxygenase-1 (HO-1) defence system efficiently prevented noise-associated cardiovascular damage (Bayo Jimenez et al., 2021[6]), indicating that dietary interventions via Nrf2 activators such as sulforaphane (active nutraceutical in broccoli sprouts) may mitigate noise-mediated adverse health effects.

Collectively, these data delineate a coherent brain-vessel axis in which aircraft noise, especially at night, activates HPA/SNS pathways, drives NOX-2-dependent oxidative inflammation, uncouples eNOS, disrupts circadian/FOXO signalling, and injures both conduit and microvascular compartments. The system exhibits no chronic exposure tolerance, pathological priming for ischemic injury, and asymmetric recovery favouring large vessels. Mechanistically anchored interventions, α/β-adrenergic blockade and α1AMPK activation, attenuate oxidative stress, inflammation and endothelial dysfunction, supporting combined exposure reduction, lifestyle targeting and NGS-guided repurposing of adrenergic/AMPK-modulating therapies as well as NOX-2/FOXO/TGF-β-focused validation studies in preclinical and early clinical settings.

## From Decibels to DNA: Noise-Induced Epigenetic Reprogramming in Cardiovascular Disease

**Figure 8:**
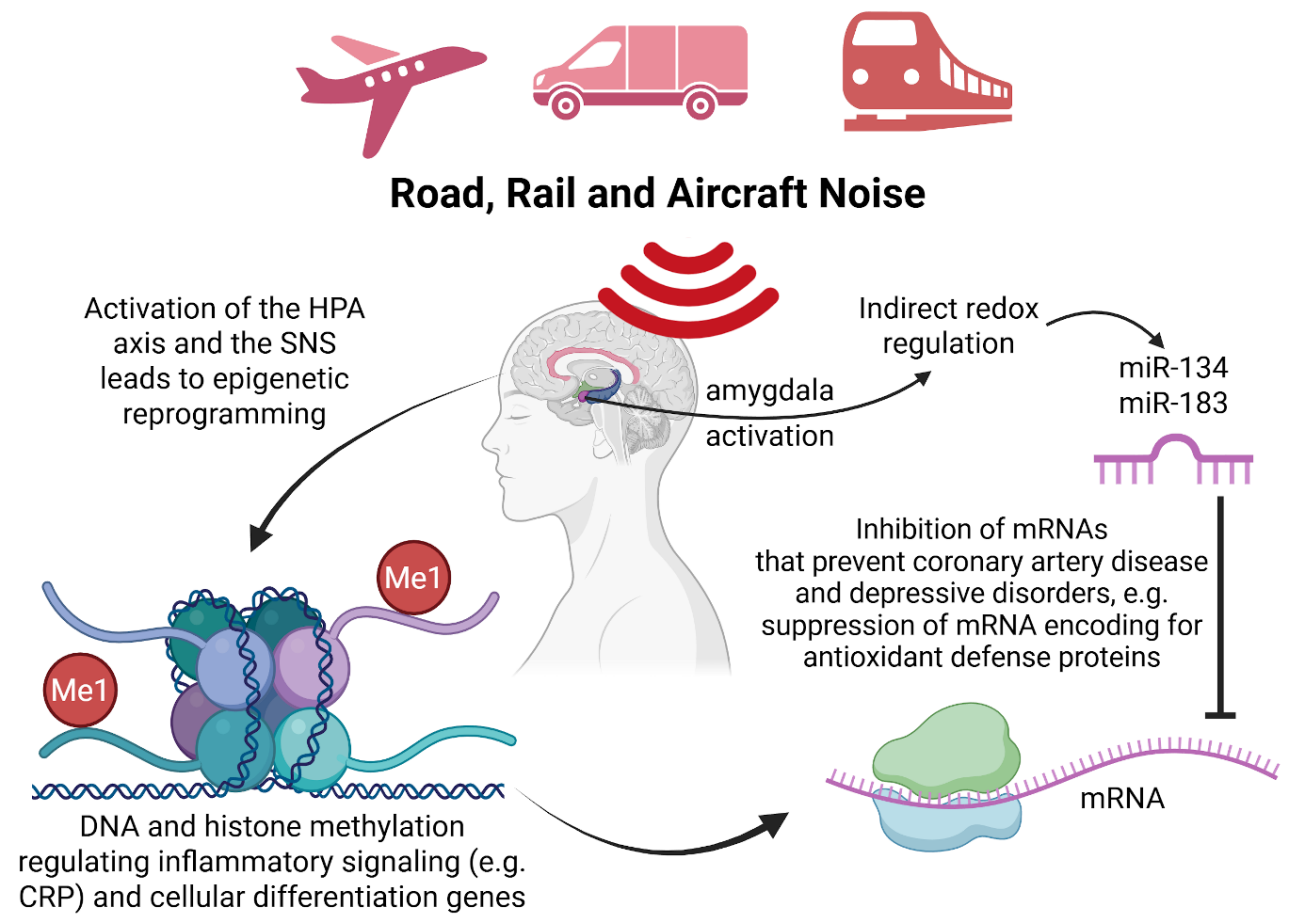
Noise-Induced Epigenetic Reprogramming in Cardiovascular Disease. Transportation noise from road, rail, and aircraft activates central stress pathways, leading to systemic and molecular effects that promote cardiovascular disease. Acoustic stimulation activates the amygdala, hypothalamic–pituitary–adrenal (HPA) axis, and sympathetic nervous system (SNS), resulting in neuroendocrine stress responses, oxidative imbalance, and epigenetic reprogramming. These responses include DNA and histone methylation (Me1), which regulate inflammatory signaling pathways (e.g. CRP) and genes involved in cellular differentiation. In parallel, indirect redox regulation alters the expression of microRNAs (miR-134, miR-183) that suppress mRNAs encoding antioxidant defense proteins and other protective targets against coronary artery disease and depressive disorders. Collectively, these changes represent redox-sensitive, stress-mediated epigenetic mechanisms that link environmental noise exposure to vascular dysfunction, inflammation, and increased cardiovascular risk (designed with Biorender).

Epigenetic mechanisms are increasingly recognized as key modulators of the initiation, progression, and severity of cardiovascular disease through their ability to regulate atherosclerotic processes and inflammatory pathways (Kuznetsova et al., 2020[60], Ordovas and Smith, 2010[87]). These processes are tightly controlled by redox signaling, and thus, noise-induced oxidative stress is likely to alter the epigenetic landscape at multiple regulatory levels (Kietzmann et al., 2017[51], Leisegang et al., 2018[64], Mikhed et al., 2015[72]). Noise exposure triggers redox imbalance and neuroendocrine stress responses that can influence DNA methylation, histone modification, and non-coding RNA regulation. Using next-generation sequencing, we and others have reported noise-induced alterations in coding RNA expression in models exploring the non-auditory effects of environmental noise (Kroller-Schon et al., 2018[55], Munzel et al., 2017[76]), as well as in studies of auditory damage and hearing loss (Lavinsky et al., 2021[63], Wei et al., 2020[123]). Importantly, noise, sleep deprivation, and mental stress can also modify the expression of non-coding RNAs, particularly microRNAs (miRNAs), which play essential roles in cardiovascular regulation (Miguel et al., 2018[70], 2020[71]) (Figure 8(Fig. 8)). Dysregulation of miRNAs may occur through indirect stress-mediated pathways, such as activation of the sympathetic nervous system and hypothalamic-pituitary-adrenal axis, or through direct mechanical injury to the inner ear during noise-induced hearing loss (Miguel et al., 2018[70]). For example, acute stress exposure has been shown to increase the expression of miR-134 and miR-183 in the central amygdala, molecules implicated both in coronary artery disease and depressive disorders (Meerson et al., 2010[69]). Several of these stress-responsive miRNAs are regulated by oxidative stress or, conversely, modulate the transcription of genes involved in antioxidant defense and pro-oxidative enzyme expression (Miguel et al., 2018[70], 2020[71]). This bidirectional interaction suggests that miRNAs serve as redox-sensitive epigenetic switches, linking environmental stressors such as noise to vascular dysfunction and inflammation. In addition to miRNA regulation, DNA methylation represents another crucial epigenetic process influencing cardiovascular risk (Greco and Condorelli, 2015[33]) (Figure 8(Fig. 8)). The DNA methylome, i.e. the overall pattern of methylated bases that modulate gene transcription, has been shown to undergo significant alterations in animal models exposed to chronic environmental noise. For instance, noise-exposed rats exhibited widespread DNA methylation changes in the brain, indicating that metabolic and stress-regulated pathways are epigenetically reprogrammed under chronic acoustic stress (Guo et al., 2017[34]). Human data support these experimental findings. In the SAPALDIA cohort study conducted in Switzerland, long-term exposure to transportation noise was associated with distinct DNA methylation patterns in peripheral blood cells, suggesting activation of inflammatory signaling, changes in cellular differentiation (e.g. via CRP), and modulation of immune responses (Eze et al., 2020[25]). Comprehensive reviews summarize these findings, showing that both auditory and non-auditory models of noise exposure produce reproducible epigenetic effects at the level of DNA, histones, and non-coding RNAs (Leso et al., 2020[65]). Also histone modifications such as lysine methylation or acetylation by noise exposure are highly likely but so far not reported, although there is clear evidence for histone modifications by other environmental stressors (Munzel et al., 2023[82]). Histone modifications are controlled by redox-sensitive methyl transferases or demethylases as well as acetyl transferases and deacetylases (e.g. sirtuins). Collectively, the available evidence indicates that epigenetic reprogramming represents a central mechanism by which environmental noise, through oxidative and psychosocial stress, contributes to the long-term cardiovascular and neurobiological consequences of exposure.

## Double Trouble: When Noise and Air Pollution Collide

Noise rarely occurs in isolation. In cities, transportation noise is almost always accompanied by air pollution, making it crucial to disentangle their individual and combined cardiovascular effects. Large-scale cohorts such as the UK Biobank and the Danish National Cohort show that noise-CVD associations persist even after rigorous adjustment for PM2.5, indicating independent impacts of noise (Liu et al., 2023[67], Roswall et al., 2021[96]).

**Figure 9:**
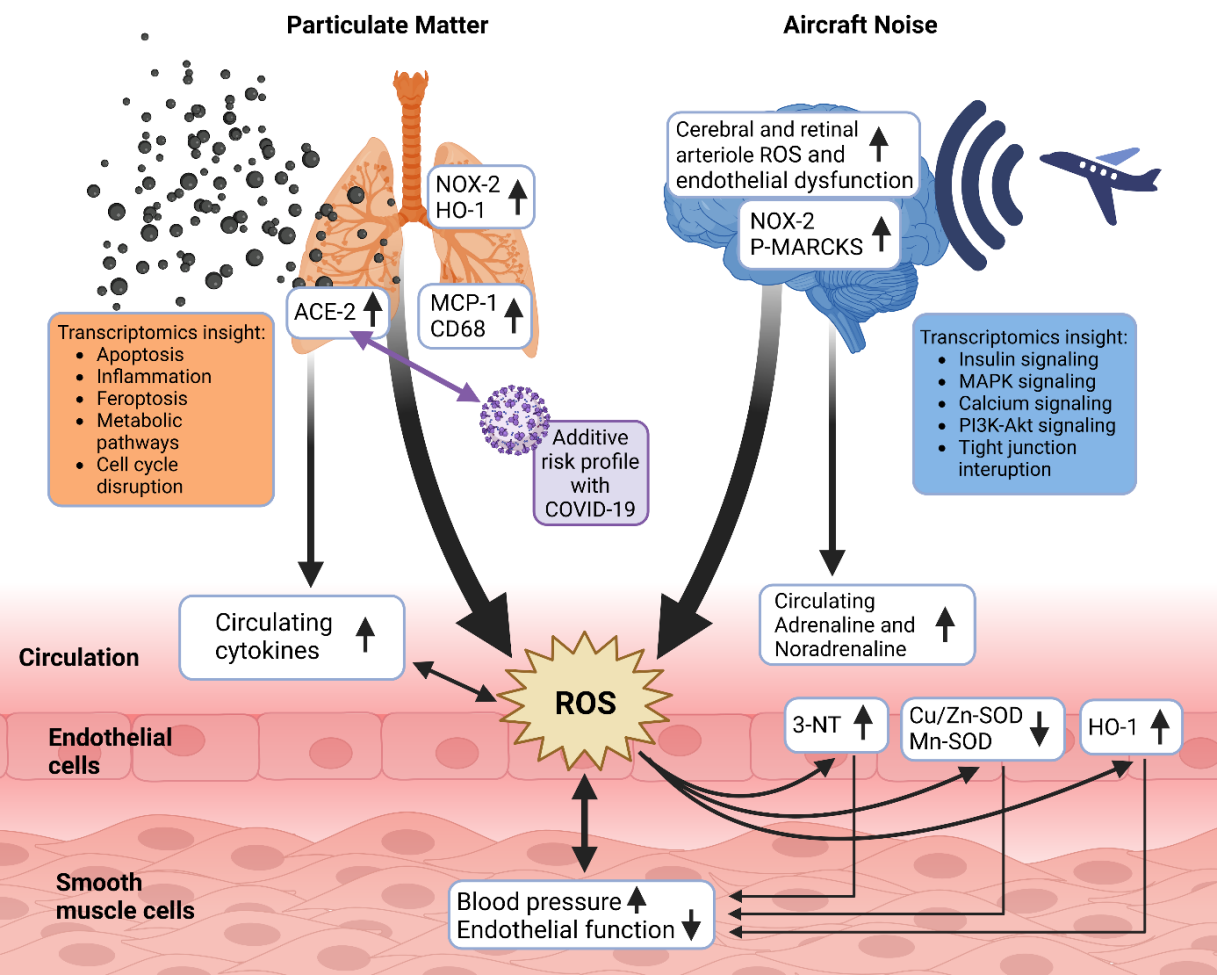
Shared and distinct pathways linking particulate matter and aircraft noise to cardiovascular dysfunction. Particulate matter exposure (left) increases pulmonary oxidative stress (NOX-2, HO-1), inflammation (MCP-1, CD68), and ACE-2 expression, leading to the release of circulating cytokines and downstream vascular effects. Aircraft noise exposure (right) induces endothelial dysfunction in cerebral and retinal arterioles via NOX-2 and P-MARCKS activation, triggering sympathetic activation and increased circulating catecholamines (adrenaline, noradrenaline). Both exposures converge on excessive formation of reactive oxygen species (ROS), which impair endothelial function, increase blood pressure, and activate oxidative stress markers (3-NT, Cu/Zn-SOD, Mn-SOD, HO-1). Transcriptomic analyses reveal distinct pathway signatures: apoptosis, inflammation, ferroptosis, metabolic pathways, and cell cycle disruption in particulate matter exposure, versus insulin signaling, MAPK signaling, calcium signaling, PI3K–Akt signaling, and tight junction disruption in noise exposure. Additive risk with COVID-19 is mediated by enhanced ACE-2 expression and systemic inflammatory activation. Collectively, particulate matter and aircraft noise share ROS-driven mechanisms that compromise vascular homeostasis and promote cardiovascular disease. Reused from (Kuntic et al., 2023) with permission.

Animal data provide strong mechanistic support. In a key experiment, Kuntic and colleagues co-exposed mice to aircraft noise and PM2.5 particles, mimicking real-world conditions. The combination produced additive to synergistic harm: endothelial dysfunction worsened, oxidative stress and inflammation rose, vascular monocyte infiltration increased, and NOX2-driven eNOS uncoupling severely impaired ^•^NO signaling (Kuntic et al., 2023[58]). These findings highlight how co-exposures intensify pathophysiological cascades (Figure 9(Fig. 9); Reference in Figure 9: Kuntic et al., 2023[58]). Overall, exposure to PM2.5 primarily causes damage in the lung, whereas noise exposure induces neuroinflammation, cerebral oxidative stress and release of stress hormones, and both damage pathways converge at the level of other organs such as the heart and the vessels.

This aligns with the broader “exposome” concept, which captures the cumulative, lifelong burden of environmental stressors (Munzel et al., 2023[82], Wild, 2005[126]). By applying such a framework, researchers and policymakers can identify high-risk groups, design effective interventions, and appreciate how overlapping exposures accelerate cardiovascular disease. Beyond CVD, both noise and air pollution contribute to diabetes, stroke, and mental health disorders, with vulnerable groups, children, the elderly, and patients with pre-existing CVD, facing the greatest risk.

## Unequal Burden: Noise across Lifespan and Vulnerable Groups

Environmental noise does not affect all populations equally. People with fewer resources, limited housing options, and less political voice are more likely to live along highways, railways, or under flight paths. The EEA 2025 report highlights that elderly individuals, the chronically ill, and low-income households disproportionately shoulder Europe's noise exposure (EEA, 2025[21]).

Critical life stages amplify risk. In children, chronic school noise has been tied to cognitive delays, impaired memory consolidation, elevated cortisol, and disrupted sleep-effects that may accumulate into long-term health and behavioral problems (Stansfeld et al., 2005[110]). In patients with cardiovascular disease, diabetes, or hypertension, even modest increases in nocturnal noise trigger repeated arousals, nocturnal hypertension, reduced heart-rate variability, and impaired endothelial repair, thereby heightening vulnerability to adverse events (Hahad et al., 2020[37], Schmidt et al., 2015[98]).

Mental health adds another layer of susceptibility. People with depression, anxiety, or sleep disorders not only report greater annoyance but also mount stronger physiological stress responses to identical acoustic stimuli. At the same time, chronic noise can fuel psychiatric conditions by persistently activating the HPA axis and disrupting emotional regulation. This creates a bidirectional cycle where noise and mental illness reinforce each other.

## Silencing the Risk: Pathways to Noise Mitigation and Cardiovascular Protection

Because noise is harmful and modifiable, reducing exposure is one of the most cost-effective public health strategies. The most substantial gains come from interventions during sleep, when the biological impact is most severe.

At the source level, road traffic measures such as low-noise asphalt, quieter tires, and speed reduction zones can lower average noise levels (L_den_) by 3-7 dB(A). Aviation benefits from steeper descent trajectories, optimized flight paths, and bans on night flights (22:00-06:00), while quieter jet engines further reduce noise footprints. On railways, composite brake pads, track grinding, and curfews on freight traffic have demonstrated success (EEA, 2025[21]).

At the transmission and receiver level, barriers, façade insulation, window glazing, and orienting bedrooms away from traffic can cut indoor nighttime noise by 10-20 dB(A). Urban planning that integrates green infrastructure, parks, tree belts, and vegetated buffers, lowers noise and simultaneously improves air quality and psychological well-being (EEA, 2025[21]).

Lifestyle and pharmacological strategies may add further protection. In animal studies, regular physical activity, intermittent fasting, and pharmacological activation of AMPK via AICAR prevented vascular dysfunction under noise exposure, highlighting potential resilience pathways (Kvandova et al., 2023[62]). While not a substitute for structural interventions, such measures could benefit high-exposure groups.

On the regulatory side, the EU Environmental Noise Directive mandates mapping and action plans but lacks binding thresholds. Aligning with WHO guidelines, particularly the ≤45 dB(A) night-time recommendation, would significantly reduce disease burden. A health equity perspective is critical: prioritizing schools, hospitals, and disadvantaged communities ensures maximal benefit while reducing inequality (EEA, 2025[21], WHO, 2018[124]).

## Beyond the Decibels: Charting the Next Frontier in Noise and Cardiovascular Research

While the evidence linking transportation noise to cardiovascular disease is now robust, major gaps remain. Longitudinal life-course studies are essential to identify windows of greatest vulnerability and to quantify cumulative exposure effects. Current metrics such as L_den_ and L_night_, though practical, may underestimate biological impact; event-based measures like NAT55 or peak sound levels (L_max_) likely better capture arousal physiology and should be integrated into future research.

Another priority is developing reliable biomarkers of both exposure and early effect. Stress hormones (cortisol), oxidative stress markers (8-isoprostane), inflammatory cytokines (IL-6), and epigenetic signatures such as DNA methylation changes hold promise for risk stratification and targeted interventions. Interventional trials are urgently needed to test whether insulation, quiet technologies, or behavioral changes translate into measurable cardiovascular benefit.

Economic evaluations add a further layer of urgency. The costs of inaction, ranging from lost productivity to rising healthcare expenditures, are high, whereas quantifying DALYs averted and QALYs gained can make the case for investment in mitigation.

Finally, noise must move from research into routine clinical care. Incorporating environmental exposure into cardiovascular risk scores, electronic health records, and patient counselling will allow clinicians to recognize and act on noise as a genuine, modifiable risk factor.

## Noise as the New Smoking: A Call for Cardiovascular Prevention

Transportation noise has emerged as a major, yet modifiable, environmental risk factor for CVD. Evidence from large epidemiological cohorts, controlled human exposure studies, and mechanistic animal models converges on a consistent picture: chronic noise drives oxidative stress, inflammation, endothelial dysfunction, and circadian disruption, key pathways in cardiovascular and metabolic pathophysiology.

The burden is not equally shared. Vulnerable groups, including the elderly, the chronically ill, and socioeconomically disadvantaged populations, face disproportionate exposure. Noise also frequently overlaps with air pollution, amplifying harm. Crucially, effective mitigation strategies exist, from source reduction and urban design to building insulation, and their implementation, especially at night, can yield substantial health gains at relatively low cost.

Given the strength of the evidence, environmental noise deserves recognition as a cardiovascular risk factor on par with hypertension, diabetes, hyperlipidaemia, and smoking. Moving forward, integration into preventive cardiology, clinical risk scores, public health guidelines, and exposome-based research frameworks is imperative. Achieving this requires close collaboration between health professionals, policymakers, and urban planners to ensure quieter, healthier environments for all.

## Notes

Thomas Münzel, Marin Kuntic, Mette Sørensen, and Andreas Daiber contributed equally and should be considered as joint first and last authors.

## Declaration

### Acknowledgments

The Mainzer Wissenschaftsstiftung supports T.M., who is also a P.I. of the German Cardiovascular Research Centre (DZHK), Partner Site Rhine Main. The work was also supported by the environmental network EXPOHEALTH funded by the state Rhineland-Palatinate, Germany. The present MS is supported by the European Union (Marko-polo Grant Agreement Number 101156161).

### Conflict of interest

All authors have no conflict of interest concerning this manuscript.

### Artificial Intelligence (AI) - Assisted Technology

AI was used for the graphical abstract: DALL-E and Midjourney.

### Author contributions

T.M. (Thomas Münzel) contributed to the conception, design, literature review, data interpretation, drafting of the manuscript, figure preparation, critical revision, and final approval of the version to be published.

A.D. (Andreas Daiber) contributed to conceptualization, mechanistic interpretation, literature review, figure development, drafting and revising the manuscript, and final approval.

M.K. (Marin Kuntic) contributed to mechanistic data integration, literature review, visualization, manuscript drafting, and critical revision for intellectual content.

M.S. (Mette Sørensen) contributed to epidemiological synthesis, integration of population-level data, critical review of mechanistic evidence, and revision of the manuscript.

M.M. (Michael Molitor) contributed to data collection, literature search, figure preparation, and manuscript editing.

All authors contributed to writing, revising, and approved the final manuscript.

## Figures and Tables

**Figure 1:**
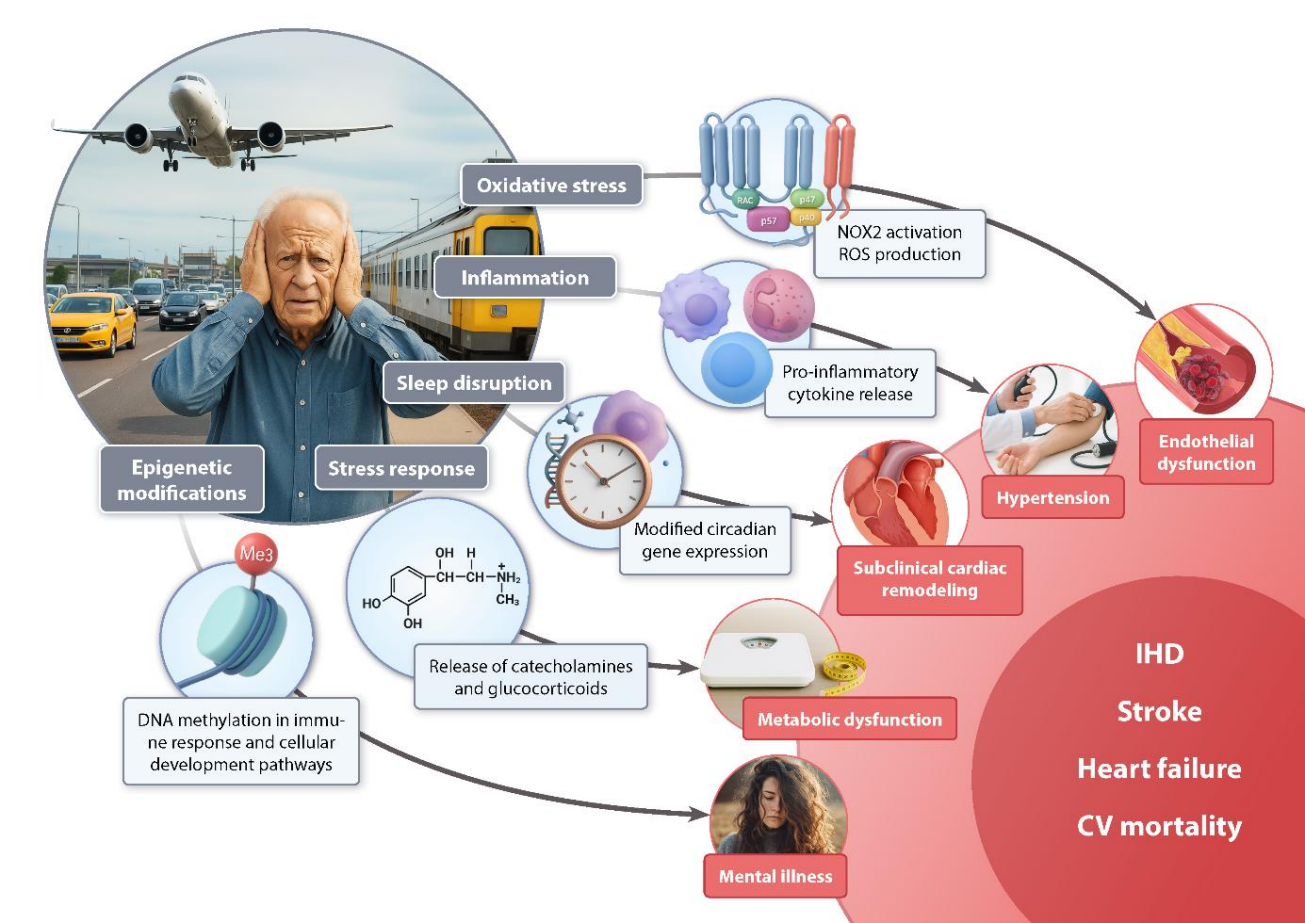
Graphical Abstract. Environmental noise triggers a cascade of biological stress responses that link chronic exposure to cardiovascular disease. Noise-induced activation of the brain’s stress pathways leads to the release of catecholamines and glucocorticoids, oxidative stress, inflammation, and endothelial dysfunction. These mechanisms are further amplified by sleep disruption, circadian rhythm disturbance, and epigenetic modifications. The resulting vascular injury promotes hypertension, cardiac remodeling, and metabolic dysfunction, ultimately contributing to ischemic heart disease, stroke, heart failure, and increased cardiovascular mortality.
